# An updated protocol for a systematic review of implementation-related measures

**DOI:** 10.1186/s13643-018-0728-3

**Published:** 2018-04-25

**Authors:** Cara C. Lewis, Kayne D. Mettert, Caitlin N. Dorsey, Ruben G. Martinez, Bryan J. Weiner, Elspeth Nolen, Cameo Stanick, Heather Halko, Byron J. Powell

**Affiliations:** 1 Kaiser Permanente Washington Health Research Institute, MacColl Center for Health Care Innovation, 1730 Minor Avenue, Suite 1600, Seattle, WA 98101 USA; 2 Department of Psychological and Brain Sciences, Indiana University, 1101 E 10th Street, Bloomington, IN 47405 USA; 3 Department of Psychiatry and Behavioral Sciences, Harborview Medical Center, University of Washington, 325 9th Ave, Box 354946, Seattle, WA 98104 USA; 4 Psychology Department, Virginia Commonwealth University, 806 W. Franklin St, Box 842018, Richmond, VA 23284 USA; 5 Department of Global Health, University of Washington, 1510 San Juan Road, Box 357965, Seattle, WA 98195 USA; 6 Hathaway-Sycamores Child and Family Services, 210 S DeLacey Ave, Suite 110, Pasadena, CA 91105-2074 USA; 7 Department of Psychology, University of Montana, 32 Campus Drive, Missoula, MT 59812 USA; 8 Department of Health Policy and Management, Gillings School of Global Public Health, University of North Carolina at Chapel Hill, 135 Dauer Drive, Chapel Hill, NC 27599 USA

**Keywords:** Systematic review, Implementation, Dissemination, Instruments, Measures, Evidence-based assessment, Psychometrics, Pragmatic

## Abstract

**Background:**

Implementation science is the study of strategies used to integrate evidence-based practices into real-world settings (Eccles and Mittman, Implement Sci. 1(1):1, 2006). Central to the identification of replicable, feasible, and effective implementation strategies is the ability to assess the impact of contextual constructs and intervention characteristics that may influence implementation, but several measurement issues make this work quite difficult. For instance, it is unclear which constructs have no measures and which measures have any evidence of psychometric properties like reliability and validity. As part of a larger set of studies to advance implementation science measurement (Lewis et al., Implement Sci. 10:102, 2015), we will complete systematic reviews of measures that map onto the Consolidated Framework for Implementation Research (Damschroder et al., Implement Sci. 4:50, 2009) and the Implementation Outcomes Framework (Proctor et al., Adm Policy Ment Health. 38(2):65-76, 2011), the protocol for which is described in this manuscript.

**Methods:**

Our primary databases will be PubMed and Embase. Our search strings will be comprised of five levels: (1) the outcome or construct term; (2) terms for *measure*; (3) terms for *evidence-based practice*; (4) terms for *implementation*; and (5) terms for *mental health*. Two trained research specialists will independently review all titles and abstracts followed by full-text review for inclusion. The research specialists will then conduct measure-forward searches using the “cited by” function to identify all published empirical studies using each measure. The measure and associated publications will be compiled in a packet for data extraction. Data relevant to our Psychometric and Pragmatic Evidence Rating Scale (PAPERS) will be independently extracted and then rated using a worst score counts methodology reflecting “poor” to “excellent” evidence.

**Discussion:**

We will build a centralized, accessible, searchable repository through which researchers, practitioners, and other stakeholders can identify psychometrically and pragmatically strong measures of implementation contexts, processes, and outcomes. By facilitating the employment of psychometrically and pragmatically strong measures identified through this systematic review, the repository would enhance the cumulativeness, reproducibility, and applicability of research findings in the rapidly growing field of implementation science.

**Electronic supplementary material:**

The online version of this article (10.1186/s13643-018-0728-3) contains supplementary material, which is available to authorized users.

## Background

Implementation science—the study of strategies used to integrate evidence-based practices into real-world settings [1]—is a rapidly growing, transdisciplinary field with enormous potential to change the way that mental health and health services are delivered in community settings [2]. Central to the identification of replicable, feasible, and effective implementation strategies is the ability to assess the impact of contextual constructs and intervention characteristics that may influence implementation [3]. Unfortunately, measurement of implementation-relevant constructs and outcomes has been undermined by a number of methodological and conceptual issues, making it difficult to understand what measures to use to assess what variables, when, and at what level of analysis [4].

One specific issue is the synonymy, homonymy, and instability of constructs [5]. In this measurement context, synonymy can refer to measures with similar items that purport to assess different constructs. Homonymy can refer to measures that purport to assess the same construct, but do so with dissimilar items. Instability can refer to the unpredictable adapting and shifting of measures’ content over time [5]. A second measurement issue stems from the similarities, redundancies, and differences in theories, models, and frameworks [6]. It is difficult to standardize optimal measurement practices with a variable and inconsistent theoretical infrastructure and associated terms and definitions. A third measurement issue comes from the lack of clarity regarding the appropriate measurement method (i.e., latent versus objective or manifest) for the target level of analysis [7], which may lead to an overreliance on and inappropriate use of self-report measures [4]. One final measurement issue is the preponderance of home-grown measures (often subject to a one-time use phenomenon) with largely unknown and unexamined psychometric properties (e.g., reliability and validity; [7]). All of these issues threaten the extent to which the field’s measures exhibit basic psychometric properties such as reliability and validity. Of greatest concern is that without reliable and valid measurement scientific findings are limited in their interpretability, comparability, and generalizability [4, 8].

Over the past decade, seven systematic reviews of contextual factors have been conducted in the field of implementation science.^1^ Although these systematic reviews have been critical to synthesizing the knowledge base, many gaps remain. Three of these reviews focused only on a single construct (e.g., fidelity and clinician behavior [10, 11]; organizational readiness to change [12]), one on five key organization-level constructs (leadership, vision, managerial relations, climate, and absorptive capacity [13]), and two on numerous constructs depicted in a model that was rather limited in scope (Framework for Effective Implementation [14]; Theoretical Framework for 27 Predictors of Adoption [15]). Only two studies evaluated measures’ content validity and found that 56 and 58% of the measures, respectively, had established content validity evidence [11, 12], indicating that nearly half of the included measures had not been tested to ensure that the items represented all facets of a known construct. More commonly, these studies reported on the psychometric evidence of measures in broad terms by simply indicating whether or not reliability or validity was assessed (or whether or not information about reliability or validity was available). With such limited information regarding psychometric quality, the impact of these reviews is undermined.

The Society for Implementation Research and Collaboration^2^ (SIRC) prioritized an initiative to aid researchers in identifying and selecting measures for key constructs by increasing accessibility of measures and information regarding their psychometric evidence. With in-kind support, SIRC developed a methodology and published preliminary results of an enhanced systematic review of measures that assessed constructs delineated in the (1) Consolidated Framework for Implementation Research (CFIR [16]) [3] and (2) Implementation Outcomes Framework (IOF; [2, 17]); (see Lewis et al. [3]). Across the 34 constructs, SIRC identified 420+ measures, revealing several constructs for which no measures exist and others for which 20+ measures were available. Of the existing measures, authors of only 71% of studies had defined the construct of interest, suggesting that content validity might be compromised. Moreover, SIRC coded studies to characterize the measure development process and found that, on average, researchers did not even pass through three of eight possible gold standard phases.^3^ This preliminary research informed a series of measures- and methods-focused studies that were funded by the National Institute of Mental Health with the long-term objective of making available a comprehensive battery of reliable, valid, and pragmatic measures that researchers and stakeholders could use to advance implementation science and practice [19].

This manuscript provides a detailed account of the systematic review protocol used to advance the aim: identify CFIR and IOF-linked measures that demonstrate both psychometric and pragmatic evidence. Although development of the pragmatic construct and its associated rating criteria is currently underway [20, 21], we have made substantial revisions to the SIRC-established systematic review methodology and the approach to psychometric evaluation [3]. These changes were made because funding allowed us to expand the scope of our approach. Rating, for instance, was expanded by applying each rating criterion to each measure subscale, by including additional criteria (i.e., convergent validity, discriminant validity, concurrent validity, known groups validity), by specifying which implementation outcome was the target in tests of predictive validity, and by engaging in a more comprehensive approach to measure-construct mapping. We also brought content and methods experts onto the team, which informed selection of new databases and a new set of search strings. Finally, we vetted our draft protocol with international advisors who suggested changes to our evidence-based assessment criteria (e.g., include an anchor [− 1] that reflects evidence of poor psychometric evidence). The protocol described herein addresses pressing gaps in the literature, the results of which will inform a measurement-focused research agenda for the field of implementation science.

## Methods

### Guiding frameworks

Over 60 theories, frameworks, and models for dissemination and implementation now exist [6]. The CFIR and IOF were selected because together they are the most widely accessed frameworks guiding the constructs and outcomes for evaluation and arguably are most comprehensive. Our team conceptualizes the CFIR as including putative predictors, moderators, and mediators of implementation success, whereas the IOF highlights the salient implementation outcomes that are distinct from service and client outcomes [17]. Including CFIR sub-constructs, our protocol will be applied to identify measures of 47 unique constructs and outcomes following PRISMA guidelines; see Additional file 1 for PRISMA Checklist. This project was not registered with PROSPERO.

### Search strategy

To identify CFIR- and IOF-related measures, we will access two widely used bibliographic databases: (1) PubMed will allow for automatic inclusion of synonyms and Medical Subject Headings, and (2) Embase will maximize our ability to capture international content, spanning biomedical journals from 90 countries [22]. We will generate our search strings in consultation with PubMed specialists and a library scientist, acknowledging the unique issues that emerge when searching for measures (e.g., imperfect database indexing, variable terminology, lack of reporting in titles, and abstracts [23]). Our final search string will be comprised of five levels: (1) the outcome or construct term (e.g., *sustainability*) and synonymous or relevant terms (e.g., *maintenance*, *institutionalization*, *routinization*); (2) terms for *measure* (e.g., *instrument*, *survey*, *questionnaire*); (3) terms for *evidence-based practice* (e.g., *innovation*, *guideline*, *empirically supported treatment*); (4) terms for *implementation* (e.g., *diffusion*, *knowledge translation*, *adoption*); and (5) terms for *mental health* (e.g., *behavioral health*, *mental disease*, *psychiatry*). Search parameters will also be set to identify those written in English, published in peer-reviewed journals, and published from 1985 onwards. We chose to limit our search to articles published after 1985 because implementation science literature did not begin to take shape until the late 1980s.

### Inclusion/exclusion criteria

The following inclusion/exclusion criteria will be applied to both the title and abstract review and full-text review; see Table 1 for an overview of these criteria. First, in line with the aim to characterize the quality of implementation-related measures in the mental or behavioral health space (per our funding source), we will only include studies that implement behavioral health interventions (e.g., cognitive behavioral therapy) and/or assess behavioral health outcomes (e.g., depression, substance use). Second, to fit our scope, measures (and associated articles) will need to demonstrate relevance to implementation, which is defined as the process of integrating evidence-based practices into a community setting [24]. For example, a study evaluating organizational capacity for implementing an evidence-based practice would fit within our scope, while an effectiveness trial of an evidence-based practice would not. Third, consistent with our focus on quantitative measures (e.g., self-report surveys, formulas, equations), qualitative methods will be excluded. In the title and abstract screening phase, two trained research specialists will apply these inclusion/exclusion criteria independently and meet to reach consensus on which articles hold potential of including a quantitative measure of an implementation-relevant construct that can be used in the behavioral health context. Any article including one or more such measures would advance to the full-text review; that is, in order to ensure the highest yield of unique measures, articles need not be focused on measure development to be included. In the full text screening phase, we will employ a hierarchical exclusion method, excluding first on the behavioral health criterion, next on the implementation criterion, and finally on the quantitative criterion. This hierarchical exclusion process will be applied independently by the two coders who will meet to resolve discrepancies.

**Table 1:** Inclusion and Exclusion Criteria

Domain	From inclusion/exclusion criteria
Intervention	Include: • Behavioral health interventions broadly construed, typically these are psychosocial interventions (e.g., cognitive behavioral therapy, motivational interviewing, multisystemic therapy) • Behavioral health interventions could also include care coordination, case management, and screening Exclude: • Physical health interventions (e.g., surgery)
Outcomes	Include: • Behavioral health-relevant outcomes include but are not limited to mental health (e.g., depression, anxiety, trauma), substance use, and social and role functioning Exclude: • Physical health outcomes (e.g., blood pressure)
Setting	Include: • Behavioral health-friendly settings include but are not limited to mental health treatment centers, medical care facilities in which behavioral health is integrated, criminal justice, education, and social service Exclude: • N/A
Measurement type	Include: • Quantitative measures, typically self-report surveys, formulas, and equations Exclude: • Qualitative evaluation

The final set of articles within each construct will be carefully evaluated for the existence of one or more CFIR- or IOF-related measures. We acknowledge the possibility that measures might have items or subscales that map onto several constructs, therefore we will catalog each measure in a construct assignment phase. We will utilize a two-pronged approach to construct assignment. First, we will record the author’s decision regarding which construct it purportedly measures regardless of whether a definition is provided. However, we observed in our pilot work that authors’ measure descriptions (or labels) often fail to capture the range of item content [3]. That is, an author could define a measure as an assessment of *readiness for implementation* while item content might represent constructs such as *implementation climate*, *available resources*, or *leadership engagement*. In order to account for these additional content areas, we will utilize an established item-analysis approach employed in a recent measure-focused systematic review [14]. Specifically, two trained research specialists will carefully review each measure’s item pool and items will be given a construct label only if two or more items are identified as assessing relevant constructs or outcomes. Research specialists will meet to discuss discrepancies and engage the principal investigator if they are unable to come to agreement. If items are not provided for coding, research specialists will map the measure more broadly to one of the five CFIR domains: *intervention characteristics*, *characteristics of individuals*, *inner setting*, *outer setting*, or *process*.

Once measures are assigned to their respective constructs and domains, we will complete measure-forward literature searches in PubMed and Embase bibliographic databases utilizing the “cited by” function. We will start by searching for the article that describes the measure’s development or the first article detailing the measure’s empirical use (i.e., the “source” article), as not all articles have an associated formal measure development study. Once locating the measure’s source article, we will engage the “cited by” search feature to yield a preliminary list of articles to be reviewed. Measures with published names (e.g. Evidence-Based Practice Scale, EBPAS [25]) will also be sought by entering the full name of the measure in quotation marks into the database search field. Once complete, research specialists will review the articles to include only those that used the measure in an empirical study in a behavioral health context. Articles will be excluded in this phase if they merely mention the measure or source article citation but do not use it (e.g., citation used in background or discussion sections) or do not present unique data on its use. After all relevant literature is retrieved, articles will be compiled into measure packets. Measures that have been adapted such that only their referent has been changed will be included in the psychometric assessment with the original version of the measure. For example, if the original measure says “children’s mental health clinic” and the author replaces with “community health center” the two studies will be included in the same psychometric evaluation. If the author changes any other word(s) other than the referent, it will be excluded from that measure’s psychometric evaluation. We will report the frequency of adaptions made for any given measure.

### Psychometric and Pragmatic Evidence Rating Scale (PAPERS)

The Psychometric and Pragmatic Evidence Rating Scale (PAPERS) criteria are based on our previous measurement work [3]. The original rating scale included six criteria that assessed only psychometric properties: reliability (i.e., internal consistency), criterion validity (i.e., predictive validity), dimensionality (i.e., structural validity), responsiveness (i.e., sensitivity to change), norms (i.e., mean and standard deviation), and usability (i.e., number of items). The current review will take a more expansive and comprehensive approach to evaluating measures. We are currently in the process of developing a pragmatic evidence rating scale for measures to be used in implementation evaluations. We are using a multi-phased stakeholder-driven approach to developing these criteria with preliminary results available suggesting the importance of the following domains: acceptable, compatible, easy, and useful. For the psychometric evidence evaluation, we added three criteria to assess construct validity (i.e., discriminant, convergent, and known-groups validity). Nine psychometric properties are included in our final rating scale: internal consistency, norms, and responsiveness, as well as convergent, discriminant, known-groups, structural, predictive, and concurrent validity. Once the pragmatic evidence rating scale is completed, we will integrate it with the psychometric evidence rating scale to form PAPERS for comprehensive evaluation of implementation measures. A detailed description of the psychometric properties follows; see Table 2 for an overview of the criteria and their definitions.

**Table 2:** Overview of psychometric rating criteria

Internal consistency	Indicates whether several items that purport to measure the same construct actually produce similar scores in the same test [26].
Convergent validity	Defined as the degree to which two constructs that are theoretically related are in fact related [26].
Discriminant (divergent) validity	Measures the degree to which two constructs that are theoretically distinct are in fact distinct [26].
Known-groups validity	Investigates whether distinct groups with differing characteristics can be differentiated [27].
Structural validity	Refers to the degree to which all test items rise and fall together, otherwise known as “test structure” [28].
Predictive validity	Refers to the degree to which a measure can predict or correlate with an outcome of interest measured at some point in the future [29]
Concurrent validity	Assesses whether two measurements taken at the same time are correlated, and the measure under consideration is compared to an established measure of the same construct [29].
Responsiveness	Captures the ability of a measure to detect clinically important changes in the construct it measures over time [26]
Norms	Measured by sample size, means, and standard deviations of measures and are meant to assess generalizability.

Internal consistency indicates whether several items that purport to measure the same construct actually produce similar scores in the same test [26]. Convergent validity is defined as the degree to which two constructs that are theoretically related are in fact related [26]. Conversely, discriminant (or divergent) validity measures the degree to which two constructs that are theoretically distinct are in fact distinct [26]. Known-groups validity investigates whether distinct groups with differing characteristics can be differentiated [27]. Structural (or construct) validity refers to the degree to which all test items rise and fall together, otherwise known as “test structure” [28]. Predictive validity refers to the degree to which a measure can predict or correlate with an outcome of interest measured at some point in the future [29]. In this protocol, we will identify whether the measure demonstrates predictive validity for each of the eight IOF outcomes [17] (acceptability, feasibility, appropriateness, adoption, penetration, cost, fidelity, sustainability). Like predictive validity, concurrent validity is meant to assess if two measurements are correlated: however, the measurements are taken at the same time and the measure under consideration is compared to an established measure of the same construct [29]. Norms are measured by the sample size, means, and standard deviations of measures and are meant to assess generalizability. Responsiveness captures the ability of a measure to detect clinically important changes in the construct it measures over time [26].

Evidence of the aforementioned properties will be extracted from each measure packet to inform criterion-specific and overall scores (process described below in the “data extraction” section). Although the previous version of our rating scale contained a 5-point anchor system ranging from 0 (no evidence) to 4 (excellent evidence), we adopted a − 1 or *poor* anchor for measures that have been tested and demonstrate poor performance on a particular psychometric property. This will allow for more nuanced reporting of the existing evidence. For example, in an assessment of a measure’s predictive validity, a null or even negative association may result in a *poor* rating. Moving forward, we will also subject each measure subscale to the full set of criteria given that our pilot work revealed many researchers have used only a single subscale of a given measure. Finally, revisions were made in an effort to capture additional statistical analyses and reporting norms in the field to obtain a broader understanding of the state of the literature. In consultation with statisticians and psychometricians, we have integrated other fit indices or metrics into the revised criteria. A final version of the psychometric evidence rating scale can be found in Additional file 2.

### Data extraction and analysis methods

Two trained research specialists will independently extract data on psychometrically relevant information within each measure packet. Specifically, we will locate all information pertaining to the nine psychometric properties found in the PAPERS criteria described above. To do this, research specialists will be trained by the principal investigator in measure development, psychometric properties, and the specific PAPERS criteria. Research specialists will read articles generated by the study team (e.g., [3, 4, 7, 19]), related measure evaluation systems (e.g., COSMIN [30]), and implementation-science-focused systematic reviews of measures [14, 15]. Research specialists will then review the project manual and complete two highlighting tests to demonstrate content mastery. First, research specialists will receive blank measure packets to practice extracting data relevant to the nine criteria. These packets will be checked by the principal investigator, and once discrepancies are resolved (if any), research specialists will complete a second highlighting test. The second test will be a packet that contains both errors of omission (e.g., information relevant to structural validity was missed) and commission (information was highlighted and tagged as being relevant to structural validity when it is not). Once research specialists pass both tests (with 90% or higher), they will be permitted to independently extract data.

Once data are extracted from the packets, they will be analyzed per the nine psychometric evidence rating scale criteria. Each packet will be reviewed by two trained research specialists, who will work independently to complete the rating phase. As noted above, the rating scale for each criterion is “poor” (− 1), “none” (0), “minimal/emerging” (1), “adequate” (2), “good” (3), or “excellent” (4), with the specifics of the rating scale for each criterion located in Additional file 2. A “worst score counts” methodology will be applied for the rating of the packets [30]. That is, if in one study, a measure demonstrated excellent evidence equivalent to a “4” for internal consistency, but exhibited only adequate evidence equivalent to a “2” in another study, the rater would assign a final internal consistency rating of “2” for that specific measure. Rating discrepancies of 1-point are averaged across the two raters, but discrepancies of 2 or more are discussed with the principal investigator to resolve.

To describe the overall availability and quality of psychometric evidence of the measures, simple statistics (i.e., frequencies) will be calculated. A total score is given to each measure by calculating the sum of the nine psychometric evidence rating scale criteria. Bar charts will display head-to-head comparisons of the nine criteria for all the measures within the various constructs of interest. Each bar chart will provide a visual comparison of overall-measure quality between measures, evaluated through the length of the bar. Additionally, the shading of the bar charts will provide a visual comparison within-criterion to assess the evidence of each criterion for the measures. For an example of the bar chart layout, please Additional file 3.

## Discussion

This manuscript offers a detailed description of a systematic review protocol for identifying and rating quantitative measures of implementation-relevant constructs and outcomes. The strengths of this approach include its broad coverage of constructs and expansive set of rating criteria. Despite these strengths, there are two key limitations. First, our criteria favor classical test theory-informed measure development and testing. Item response theory offers a more contemporary, alternative approach to measure development that would be of benefit to implementation science, but it is not one our rating system is capable of accommodating. Second, our scope was necessarily constrained to reflect the priorities of our funding agency (the National Institute of Mental Health). Thus, although the CFIR and implementation outcomes have widespread relevance across health (and into other settings), our review will only yield measures (and associated evidence from use) in the behavioral health space. Complementary efforts to expand this work into the broader physical health space are ongoing [31].

Upon completion of the systematic review, anticipated in October 2018, we will build a centralized, accessible, searchable repository through which researchers, practitioners, and other stakeholders can identify psychometrically and pragmatically strong measures of implementation contexts, processes, and outcomes. The repository’s graphical interface will display measures’ psychometric and pragmatic ratings and facilitate head-to-head comparisons of measures of the same construct, where multiple measures exist. With information available on an expanded set of psychometric criteria, repository users can select measures with the highest ratings overall or the highest rating on a specific criterion (e.g., known-groups validity), depending on their needs. The creation of a centralized, accessible, searchable repository of psychometrically and pragmatically rated measures of core constructs in two of the most widely used conceptual frameworks in the field of implementation science is expected to curtail the proliferation of “home-grown” or one-use measures with dubious or unknown reliability, validity, and practicality. Moreover, by facilitating the employment of psychometrically and pragmatically strong measures identified through this systematic review, the repository would enhance the cumulativeness, reproducibility, and applicability of research findings in the rapidly growing field of implementation science.

## Additional files

Additional file 1: PRISMA-P 2015 Checklist: checklist adapted for use with systematic review protocol submissions to BioMed Central journals. (DOCX 36 kb)

Additional file 2: Psychometric evidence rating scale: presents nine criteria by which data is extracted and rated. (DOCX 30 kb)

Additional file 3: Psychometric head-to-head comparison: example bar chart layout provided to demonstrate overall availability and quality of psychometric evidence of available measure. (DOCX 31 kb)

## Abbreviations

CFIR: Consolidated Framework for Implementation Research
EBPAS: Evidence-Based Practice Scale
IOF: Implementation Outcomes Framework
PAPERS: Psychometric and Pragmatic Evidence Rating Scale
SIRC: Society for Implementation Research and Collaboration

## Footnotes

1 See Rabin et al. [9] for a summary of these studies and related resources.

2 https://societyforimplementationresearchcollaboration.org/

3 Eight “gold standard” stages of measure development are informed by Walsh, Betz: [18]: (1) construct is defined, (2) initial items are generated by a group of experts, (3) pilot test of items with representative sample, (4) validity and reliability tests conducted based on pilot testing, (5) instrument is refined based on pilot results, (6) refined instrument is administered to the targeted sample, (7) validity and reliability tests are performed, and (8) psychometric properties are reported.
