# Supplementary material for: An updated protocol for a systematic review of implementation-related measures
Source: Syst Rev. 2018 Apr 25;7:66. doi: 10.1186/s13643-018-0728-3 (PMC5918558; doi:10.1186/s13643-018-0728-3)
Supplement: Supplementary file 2 — Psychometric evidence rating scale: presents nine criteria by which data is extracted and rated. (DOCX 30 kb) [file 13643_2018_728_MOESM2_ESM.docx]

| **Reliability – Internal Consistency** | |
| --- | --- |
| **Note: When only subscale α values are given, provide all and apply ‘worst score counts’ rule.** | |
| **-1** | Poor (P): Cronbach’s α values of **< 0.50** |
| **0** | None (N): Internal consistency measures are not applicable for this instrument **OR** classical test theory anchors are not appropriate (results reported using item response theory) **OR** α values are not yet available for the full measure scale or any associated subscales. |
| **1** | Minimal/Emerging (M): Cronbach’s α values = **0.50-0.69** |
| **2** | Adequate (A): Cronbach’s α values of = **0.70 - 0.79** |
| **3** | Good (G): Cronbach’s α values of = **0.80 - 0.89** |
| **4** | Excellent (E): Cronbach’s α values of **≥ 0.90** |
| **Construct Validity - Convergent** | |
| **-1** | Poor: **Cohen’s *d* ≤ 0.10** |
| **0** | None (N): Convergent validity measures are not applicable for this instrument OR convergent validity was not assessed. |
| **1** | Minimal/Emerging: **0.10 < Cohen’s *d* ≤ 0.20** |
| **2** | Adequate: **0.20 < Cohen’s *d* ≤ 0.50** |
| **3** | Good: **0.50 < Cohen’s *d* ≤ 0.80** |
| **4** | Excellent: **Cohen’s *d* > 0.80** |
| **NOTE: If Pearson’s *r* is given, use the effect size calculator to calculate Cohen’s *d.*** <https://www.polyu.edu.hk/mm/effectsizefaqs/calculator/calculator.html>  **Also, note that these criteria also apply to comparisons between subscales.** | |
| **Construct Validity-Discriminant** | |
| **-1** | Poor: **Cohen’s *d* > 0.80** |
| **0** | None (N): Discriminant validity measures are not applicable for this instrument OR discriminant validity was not assessed. |
| **1** | Minimal/Emerging: **0.50 < Cohen’s *d* ≤ 0.80** |
| **2** | Adequate: **0.20 < Cohen’s *d* ≤ 0.50** |
| **3** | Good: **0.10 < Cohen’s *d* ≤ 0.20** |
| **4** | Excellent: **Cohen’s *d* ≤ 0.10** |
| **NOTE: If Pearson’s *r* is given, use the effect size calculator to calculate Cohen’s *d.*** <https://www.polyu.edu.hk/mm/effectsizefaqs/calculator/calculator.html>  **Also, note that these criteria also apply to comparisons between subscales.** | |
| **Construct Validity-Known-Groups** | |
| **Categories:** Demographics, Roles/Professions, Programs/Treatments, Organizations, Intervention Conditions | |
| **-1** | Poor (P): Known-groups validity failed to be detected. |
| **0** | None (N): Known-groups validity not yet tested. |
| **1** | Minimal/Emerging (M): Statistically significant difference between groups detected, but no hypothesis tested |
| **2** | Adequate (A): Two or more statistically significant difference between groups detected, but no hypotheses tested |
| **3** | Good (G): Statistically significant difference between groups detected AND hypothesis tested |
| **4** | Excellent (E): Two or more statistically significant differences between groups detected AND hypotheses tested |
| **Criterion Validity-Predictive** | |
| Evidence of correlation (Pearson’s *r*) between instrument and scores on another test (measuring a distinct construct of interest or outcome) administered **at some point in the future.** | |
| **-1** | Poor (P): Pearson’s *r* **< 0.10** |
| **0** | None (N): Predictive validity not tested. |
| **1** | Minimal/Emerging (M): Pearson’s *r* = **0.10-0.29** |
| **2** | Adequate (A): Pearson’s *r* = **0.30-0.49** |
| **3** | Good (G): Pearson’s *r* = **0.50-0.69** |
| **4** | Excellent (E): Pearson’s *r* **> 0.70** |
| **NOTE: If unstandardized regression coefficients (betas) are reported, use the effect size calculator to translate them into Pearson’s *r* values and follow the same rules as above.**  <https://www.campbellcollaboration.org/escalc/html/EffectSizeCalculator-R7.php>  **&**  **If discriminant function analysis is reported, use the measure of variance explained. Anchors for this can be found in “Structural Validity” section.** | |
| **Criterion Validity-Concurrent** | |
| Evidence of correlation (Pearson’s *r*) between instrument and scores on another test (measuring a distinct construct of interest or outcome) administered at the **same point in time.** | |
| **-1** | Poor (P): Pearson’s *r* **< 0.10** |
| **0** | None (N): Concurrent validity not tested. |
| **1** | Minimal/Emerging (M): Pearson’s *r* = **0.10-0.29** |
| **2** | Adequate (A): Pearson’s *r* = **0.30-0.49** |
| **3** | Good (G): Pearson’s *r* = **0.50-0.69** |
| **4** | Excellent (E): Pearson’s *r* **> 0.70** |
| **NOTE: If unstandardized regression coefficients (betas) are reported, use the effect size calculator to translate them into Pearson’s *r* values and follow the same rules as above.**  <https://www.campbellcollaboration.org/escalc/html/EffectSizeCalculator-R7.php>  **&**  **If discriminant function analysis is reported, use the measure of variance explained. Anchors for this can be found in “Structural Validity” section.** | |
| **Dimensionality-Structural Validity** | |
| Normed Fit Index = NFI ; Incremental Fit Index = IFI  Goodness of Fit Index = GFI ; Tucker-Lewis Index = TLI  Comparative Fit Index = CFI ; Relative Noncentrality Fit Index = RNI  Standardized RMR = SRMR ; Root Mean Square Error of Approximation = RMSEA  Weighted Root Mean Residual = WRMR | |
| **-1** | Poor (P):  The sample consisted of less than 5 times the number of items AND exploratory factor analysis explained **< 25%** of variance **OR** |
|  | NFI **OR** IFI **OR** GFI **OR** TLI **OR** CFI **OR** RNI  **≤ 0.88**  **OR** SRMR **OR** RMSEA = **X ≥ 0.10**  **OR** WRMR **≥ 0.92** |
| **0** | None (N): No exploratory or confirmatory factor analysis has yet been performed, nor have any Item Response Theory (IRT) tests of (uni-) dimensionality have been conducted **OR** analysis has been conducted but percent variance is unexplained and cannot be calculated **OR** only principal components analysis has been conducted. |
| **1** | Minimal/Emerging (M):  The sample consisted of 5 times the number of items AND exploratory factor analysis explained **< 25%** of variance **OR** |
|  | NFI **OR** IFI **OR** GFI **OR** TLI **OR** CFI **OR** RNI = **0.88 < X ≤ 0.90**  **OR** SRMR **OR** RMSEA = **0.08 ≤ X < 0.10**  **OR** WRMR = **0.90 ≤ X < 0.92** |
| **2** | Adequate (A):  The sample consisted of 5 times the number of items but is less than 100 in total AND an exploratory factor analysis explained **< 50%** of variance **OR** |
|  | NFI **OR** IFI **OR** GFI **OR** TLI **OR** CFI **OR** RNI = **0.90 < X ≤ 0.95**  **OR** SRMR **OR** RMSEA = **0.05 ≤ X < 0.08**  **OR** WRMR = **0.85 ≤ X < 0.90** |
| **3** | Good (G):  The sample consisted of 5 times the number of items and is greater than or equal to 100 in total OR the sample consisted of 5-7 times the number of items but is less than 100 in total AND in either case exploratory  factor analysis explained  **< 50%** of variance **OR** |
|  | NFI **OR** IFI **OR** GFI **OR** TLI **OR** CFI **OR** RNI = **0.95 < X ≤ 0.97**  **OR** SRMR **OR** RMSEA = **0.03 ≤ X < 0.05**  **OR** WRMR = **0.83 ≤ X < 0.85** |
| **4** | Excellent (E):  The sample consisted of 7 times the number of items and is greater than 100 in total AND an exploratory factor analysis explained **> 50%** of variance **OR** |
|  | NFI **OR** IFI **OR** GFI **OR** TLI **OR** CFI **OR** RNI  **> 0.97**  **OR** SRMR **OR** RMSEA = **< 0.03**  **OR** WRMR **< 0.83** |
| **Note: If multiple indices are given and they fall within differing rating anchors, use the mode score (three “good” ratings, 1 excellent rating, 1 poor rating → rated as “good.”)** | |
| **Responsiveness** | |
| Standardized Response Mean = SRM | |
| **-1** | Poor (P):  SRM **< 0.10 OR**  Pearson’s *r* **< 0.10** |
| **0** | None (N): The instrument has not been administered both pre- and post- implementation to evaluate sensitivity to change. |
| **1** | Minimal/Emerging (M):  SRM = **0.10-0.19 OR**  Pearson’s *r* = **0.10-0.29** |
| **2** | Adequate (A):  SRM = **0.20-0.49 OR**  Pearson’s *r* = **0.30-0.49** |
| **3** | Good (G):  SRM = **0.50-0.79 OR**  Pearson’s *r* = **0.50-0.69** |
| **4** | Excellent (E):  SRM **> 0.80 OR**  Pearson’s *r* **> 0.70** |
| **Norms** | |
| **-1** | Poor (P): Measures of central tendency and distribution for the total score (and subscales if relevant) based only on a very small (**n < 50**) sample are available. |
| **0** | None (N): Norms not yet available. |
| **1** | Minimal/Emerging (M):  Measures of central tendency and distribution for the total score (and subscales if relevant) based only on a small (**n = 50-99**) sample are available. |
| **2** | Adequate (A): Measures of central tendency and distribution for the total score (and subscales if relevant) based only on a small (**n = 100-299**) sample are available. |
| **3** | Good (G):  Measures of central tendency and distribution for the total score (and subscales if relevant) based on a medium (**n = 300-499**) sample are available. |
| **4** | Excellent (E):  Measures of central tendency and distribution for the total score (and subscales if relevant) based on a large (**n ≥ 500**) sample are available. |
