# Supplementary material for: An updated protocol for a systematic review of implementation-related measures
Source: Syst Rev. 2018 Apr 25;7:66. doi: 10.1186/s13643-018-0728-3 (PMC5918558; doi:10.1186/s13643-018-0728-3)
Supplement: Supplementary file 3 — Psychometric head-to-head comparison: example bar chart layout provided to demonstrate overall availability and quality of psychometric evidence of available measure. (DOCX 31 kb) [file 13643_2018_728_MOESM3_ESM.docx]

**Barriers to Treatment Integrity Implementation Survey**

- Perepletchikova, F., Hilt, L. M., Chereji, E., & Kazdin, A. E. (2009). Barriers to implementing treatment integrity procedures: Survey of treatment outcome researchers. Journal of Consulting and Clinical Psychology, 77(2), 212.

**Change Process Capability Questionnaire**

- Solberg, L. I., Asche, S. E., Margolis, K. L., & Whitebird, R. R. (2008). Measuring an organization's ability to manage change: the change process capability questionnaire and its use for improving depression care. American Journal of Medical Quality, 23(3), 193-200.

**Structured Interview of Evidence Use**

- Palinkas, L. A., Garcia, A. R., Aarons, G. A., Finno-Velasquez, M., Holloway, I. W., Mackie, T. I., ... & Chamberlain, P. (2016). Measuring use of research evidence: the structured interview for evidence use. Research on social work practice, 26(5), 550-564.

**Team Fitness Test**

- Chodosh, J., Price, R. M., Cadogan, M. P., Damron‐Rodriguez, J., Osterweil, D., Czerwinski, A., ... & Frank, J. C. (2015). A Practice Improvement Education Program Using a Mentored Approach to Improve Nursing Facility Depression Care—Preliminary Data. Journal of the American Geriatrics Society, 63(11), 2395-2399.

**Salyers Barriers & Facilitators Leadership Measure**

- Salyers, M. P., Rollins, A. L., McGuire, A. B., & Gearhart, T. (2009). Barriers and facilitators in implementing illness management and recovery for consumers with severe mental illness: trainee perspectives. Administration and Policy in Mental Health and Mental Health Services Research, 36(2), 102-111.

**State health Authority Yardstick**

- Finnerty, M. T., Rapp, C. A., Bond, G. R., Lynde, D. W., Ganju, V., & Goldman, H. H. (2009). The state health authority yardstick (SHAY). Community Mental Health Journal, 45(3), 228-236.

**Implementation Leadership Scale**

- Aarons, G. A., Ehrhart, M. G., & Farahnak, L. R. (2014). The implementation leadership scale (ILS): development of a brief measure of unit level implementation leadership. Implementation Science, 9(1), 45.

**Readiness for Integrated Care Questionnaire**

- Scott, V. C., Kenworthy, T., Godly-Reynolds, E., Bastien, G., Scaccia, J., McMickens, C., ... & Wandersman, A. (2017). The Readiness for Integrated Care Questionnaire (RICQ): An Instrument to Assess Readiness to Integrate Behavioral Health and Primary Care.

**Aarons Leader Readiness Scale**

- Aarons, G. A., Ehrhart, M. G., Farahnak, L. R., & Hurlburt, M. S. (2015). Leadership and organizational change for implementation (LOCI): a randomized mixed method pilot study of a leadership and organization development intervention for evidence-based practice implementation. Implementation Science, 10(1), 11.

**Aarons Leader Support Scale**

- Aarons, G. A., Ehrhart, M. G., Farahnak, L. R., & Hurlburt, M. S. (2015). Leadership and organizational change for implementation (LOCI): a randomized mixed method pilot study of a leadership and organization development intervention for evidence-based practice implementation. Implementation Science, 10(1), 11.
